# Plakophilin-3 Is Required for Late Embryonic Amphibian Development, Exhibiting Roles in Ectodermal and Neural Tissues

**DOI:** 10.1371/journal.pone.0034342

**Published:** 2012-04-05

**Authors:** William A. Munoz, Malgorzata Kloc, Kyucheol Cho, Moonsup Lee, Ilse Hofmann, Amy Sater, Kris Vleminckx, Pierre D. McCrea

**Affiliations:** 1 Department of Biochemistry and Molecular Biology, University of Texas M.D. Anderson Cancer Center, Houston, Texas, United States of America; 2 Program in Genes and Development, University of Texas Graduate School of Biomedical Science, Houston, Texas, United States of America; 3 Department of Surgery, The Methodist Hospital Research Institute, Houston, Texas, United States of America; 4 Joint Research Division Vascular Biology of the Medical Faculty Mannheim, University of Heidelberg- DKFZ, Mannheim, Germany; 5 Biology and Biochemistry Department, University of Houston, Houston, Texas, United States of America; 6 Department for Molecular Biomedical Research, Flanders Institute for Biotechnology VIB, Ghent, Belgium; University of Colorado, Boulder, United States of America

## Abstract

The p120-catenin family has undergone a significant expansion during the evolution of vertebrates, resulting in varied functions that have yet to be discerned or fully characterized. Likewise, members of the plakophilins, a related catenin subfamily, are found throughout the cell with little known about their functions outside the desmosomal plaque. While the plakophilin-3 (Pkp3) knockout mouse resulted in skin defects, we find larger, including lethal effects following its depletion in *Xenopus.* Pkp3, unlike some other characterized catenins in amphibians, does not have significant maternal deposits of mRNA. However, during embryogenesis, two Pkp3 protein products whose temporal expression is partially complimentary become expressed. Only the smaller of these products is found in adult *Xenopus* tissues, with an expression pattern exhibiting distinctions as well as overlaps with those observed in mammalian studies. We determined that Xenopus Pkp3 depletion causes a skin fragility phenotype in keeping with the mouse knockout, but more novel, *Xenopus* tailbud embryos are hyposensitive to touch even in embryos lacking outward discernable phenotypes, and we additionally resolved disruptions in certain peripheral neural structures, altered establishment and migration of neural crest, and defects in ectodermal multiciliated cells. The use of two distinct morpholinos, as well as rescue approaches, indicated the specificity of these effects. Our results point to the requirement of Pkp3 in amphibian embryogenesis, with functional roles in a number of tissue types.

## Introduction

The plakophilins (Pkps) constitute a subfamily of the Armadillo-repeat family of proteins, which also includes the p120- and beta-catenin subfamilies [1], [2], [3], [4], [5], [6]. Analogous to the p120-subfamily's role at adherens junctions, the Pkps assist in stabilizing the desmosomal plaque [5], [7], [8]. The Pkps interact with several desmosomal components including the trans-membrane desmosomal cadherins (desmocollins and desmogleins), and with desmoplakin, thereby aiding in connecting the junction to the cytoskeleton and contributing to tissue integrity [9], [10], [11], [12], [13], [14], [15], [16], [17], [18]. While less understood, Pkps also localize to the cytoplasm, where they can be found in stress granules and other RNA-containing particles regulating translation [19], [20]. The related p120-subfamily has been shown to modulate the cytoskeleton through direct or indirect interactions with small GTPases [21], [22], [23], [24], [25], [26], [27], [28], and it has been suggested that the Pkps may act through similar mechanisms to affect cell shape [29]. Interestingly, Pkps, like other family members, are also found in the nucleus, though Pkp3 is not often detected there [2], [30], [31], [32], [33], [34]. While not fully understood, Pkp2 binds to the nuclear RNA polymerase III holoenzyme, and Pkp1 to single strand DNA [35], [36]. Other nuclear functions for the Pkps are yet to be determined. Overall, and in common with other catenins, the varied localizations and presumably functions of Pkps is suggestive of their participation in cross-talk between the plasma membrane, cytoplasm and/or nuclear compartments.

In mammalian systems Pkp3 is predominately expressed in tissues enriched for desmosomes such as in simple epithelia or the living layers of stratified epithelia and the epidermis, although not in hepatocytes [30], [37], [38]. Low levels of Pkp3 mRNA have been found in most if not all tissues of mammals. The targeted mouse knockout of Pkp3 resulted in hair shaft abnormalities, skin inflammatory responses, and disruptions of desmosome assembly in the epidermis. As no further effects were reported, Pkp3−/− desmosomal defects in skin appear to predominate in mice [39]. In pathology, Pkp3 misexpression has been associated with non-small cell lung carcinomas, squamous cell carcinomas, gastric carcinoma, breast carcinoma and adenocarcinomas [40], [41], [42], [43], [44], [45]. It has also been shown that the reduction of Pkp3 levels in HCT116 cells enhances their metastatic potential in mice [46].

To further examine the in vivo functions of Pkp3, we specifically knocked-down Pkp3 protein expression in embryos of *Xenopus laevis*. Our work here indicates that Pkp3 is developmentally essential in amphibians, and has roles including but potentially also separate from those pertaining to the desmosomal plaque.

## Materials and Methods

### *Xenopus laevis* Pkp3 cDNA isolation

A Pkp3-specific cDNA fragment from *Xenopus laevis* was identified via screening of a cDNA library (embryonic stage 30; Stratagene), using a labeled human Pkp3 cDNA fragment. Further Pkp3 sequence was obtained using 5′ RACE of mRNA extracted from *Xenopus laevis* XTC cells, and the assembled clone entered into the pGEMTeasy vector.

### RNA isolation, semi-quantitative real time PCR and RT-PCR

Following the manufacturer's instructions for Trizol (Invitrogen), total RNA was extracted from *Xenopus* embryos. DNA was removed with RQ1 DNase (Promega M610A). Approximately 2.5 µg total RNA was reverse-transcribed into cDNA pools using oligo-dT (Invitrogen 18418-012) and SuperScript II Reverse Transcriptase (Invitrogen 18064-014). cDNA was then used as template for either real time PCR with Power SYBR Green Master Mix in an Applied Biosystems 7500 Fast Real-Time PCR System or PCR amplification. To control for DNA contamination, reactions were performed in the absence of reverse transcriptase. PCR primers are as follows: ARM-F 5′-ATGGCATTTATGAACTGTTGACCGCTTT-3′, ARM-R 5′-TCTGCGTGAGTGGGTTTAAGGTGTCC-3′, HH4-F 5′-CGGGATAACATTCAGGGTA-3′, HH4-R 5′-TCCATGGCGGTAACTGTC-3′, Pkp2-F 5′-TCGGCTGTTGCTCACATGATT-3′, Pkp2-R 5′-ATTTCTCTCGCGATCTCATTTTGG-3′, ODC-F 5′-AAAAAGCATGTGCGTTGGT-3′, ODC-R 5′-ACGGCATAAAACGGAGTGA-3′.

### Whole-mount in situ RNA hybridization

Whole-mount in situ RNA hybridizations were performed as previously described [47]. Briefly, digoxigenin-labeled sense and antisense RNA probes were prepared through in vitro transcription (DIG RNA Labeling Kit, Roche 11175025910). Probes were detected with anti-digoxigenin antibody conjugated to alkaline phosphatase (Roche 11093274910) and NBT/BCIP (Roche 11697471001) was used as substrate during reactions. Sections of stained embryos were obtained using previously described methods [48].

### Histological sections

Histological sections were prepared by embedding embryos in paraplast. Sections were then obtained at 10 µm thickness and stained with hematoxylin and eosin and mounted.

### Antibody generation and immuno-blotting

Antibodies generated against the recombinant (GST-tagged) N-terminal domain of *Xenopus* Pkp3 (amino acids 1–350; PTG labs), were affinity-purified from rabbit crude serum. The *Xenopus* PKP2 antiserum was raised in guinea pigs using a cocktail consisting of three peptides (EWRSECDTRRPTL+C, RYRTASRARQNLSQQFRQDT+C, QDLHSTYKKSYKK+C) coupled to keyhole limpet hemocyanin. Other antibodies were purchased from commercial suppliers (GAPDH, Santa Cruz sc-25778; c-myc, DSHB 9E 10; skeletal muscle, DSHB 12/101; acetylated alpha-tubulin, Sigma T7451; HA, 12CA5). Embryos were harvested at the indicated developmental stages and immuno-blotting was performed following standard procedures using 1–0.2×0.2% KPL Block (KPL 71-83-00).

### Immunostaining and fluorescent imaging

Embryos were fixed in 1× MEM and 4% Formaldehyde at specified stages for 1–2 hours followed by dehydration in 100% methanol at −20°C overnight. Rehydration was done in 1× PBS with 0.1% Tween-20 (PTw) followed by blocking in 20% goat serum in PTw. Primary antibody (Myc 1∶500; Pkp3 1∶12.5; 12/101 1∶500; acetylated alpha tubulin 1∶500) incubation was preformed overnight at 4°C in 10% goat serum in PTw. Samples were washed in PTw (5× over 5 hours), and then incubated overnight with secondary antibody (Alexa-488 or -555 conjugated to anti-rabbit or mouse specific antibody) in PTw, and washed again (5× over 5 hours). For experiments involving the detection of acetylated alpha-tubulin, 25–45 overlapping images were obtained per embryo sample using a Leica DM4000B, and then photomerged using Adobe Photoshop CS4. Sub-cellular localization was assessed via confocal microscopy using an Olympus IX70 microscope and images analyzed with Fluoview 500 software.

### Antisense morpholinos

*Xenopus* Pkp3 translation blocking morpholinos (Pkp3 MO 1 5′-CTCTCTCTCTGTCCCTGAGAGGCTT-3′ and Pkp3 MO 3 5′-GCTTTAGTGTAGGCTCGGACCCCTC-3′), and a standard morpholino (5′-CCTCTTACCTCAGTTACAATTTATA-3′) were obtained from Gene Tools.

### Embryo manipulations

*Xenopus laevis* embryos were obtained, fertilized and microinjected as previously described [49], except that microinjections were performed in 5% Ficoll in 0.3× MMR (Marc's modified ringers solution), and cultured in solution for at least one hour. Embryos were then transferred to 0.3× MMR with 50 µg/mL Gentamycin for culture at 14–18°C. Phenotypes were visually scored at various embryonic stages using a standard binocular stereoscope (Olympus SZX12).

This study was carried out in strict accordance with the recommendations in the American Association for Laboratory Animal Science Learning Library. The protocol was approved by the Institutional Animal Care and Use Committee of the The University of Texas-M.D. Anderson Cancer Center (ACUF Protocol #09-93-05717). All efforts were made to minimize suffering.

### Plasmid constructs and in vitro transcription

pCS2-based plasmids harboring *Xenopus* Pkp3 fused to a carboxy-terminal HA-tag (absence or presence of 5′UTR) or an amino-terminal Myc-tag, were generated using standard recombinant techniques for subsequent production of in vitro transcribed RNAs. Using mMESSAGE SP6 kit (Ambion), capped mRNAs were synthesized in vitro following Not1 linearization. Transcripts were then acidic phenol/chloroform extracted and unincorporated nucleotides were filtered out using Sephadex G-50 Quick Spin Columns (Roche). mRNA was ethanol precipitated at −80°C. mRNA integrity was examined via agarose gel electrophoresis and ethidium bromide staining.

### Touch sensitivity assay

Embryos from stage 26–45 were subjected to a tactile stimulus. Using either gentle displacement of the incubation dish or a pipette tip, a mild stimulus was applied to the embryo and its reaction was observed. Control embryos at earlier stages are not very active, but when a tactile stimulus is applied they swim away from the stimulus.

### Electron Microscopy

Embryos were fixed in 2% formaldehyde, 3% glutaraldehyde (EM grade from Ted Pella Inc., glutaraldehyde 8% stock, 18421; formaldehyde 16% stock, 18505) in 1× phosphate buffered saline (PBS).

For transmitted electron microscopy, samples were dehydrated in ethanol at increasing concentrations, infiltrated and embedded in LX-112 medium (Epon substitute; Ladd Research Industries). Ultrathin sections (70–100 nm) were contrasted with uranyl acetate and lead citrate according to standard protocols [50]; the sections were then examined in a JEOL 100SX transmission electron microscope (JEM, Japan) at an accelerating voltage of 80 kV.

For scanning electron microscopy, fixed samples were washed with 0.1 M cacodylate buffer, pH 7.3 for 3×10 min. The samples were then post fixed with 1% cacodylate buffered osmium tetraoxide for 1 hour, washed with 0.1 M cacodylate buffer for 3×10 min, and then in distilled water, 2×5 min. The samples were sequentially treated with Millipore-filtered 1% aqueous tannic acid for 30 min in the dark, washed in distilled water 3×10 min, and then in Millipore-filtered 1% aqueous uranyl acetate for 1 hour in the dark. Samples were rinsed with distilled water for 2×5 min, and were then dehydrated with a graded series of increasing concentrations of ethanol for 5 min each. The samples were transferred to graded series of increasing concentrations of hexamethyldisilazane (HMDS) for 5 min each and air dried overnight. Samples were mounted on double-stick carbon tabs (Ted Pella. Inc., Redding, CA), which had been previously mounted on aluminum specimen mounts (Electron Microscopy Sciences). The samples were then coated under vacuum using a Balzer MED 010 evaporator (Technotrade International) with platinum alloy for a thickness of 25 nm, and immediately flash carbon coated under vacuum. The samples were transferred to a desiccator for examination at a later date. Samples were examined in a JSM-5910 scanning electron microscope (JEOL USA, Inc.) at an accelerating voltage of 5 kV.

### Statistical Analysis

Data presented is in percentages to account for differences in total numbers between each experimental condition. Graphs produced were based on the average percentages of triplicate experiments. To analyze the significance of our phenotypes, we used the Student's T-test. To obtain P-values, we hypothesized that the two groups are statistically identical (null hypothesis), and analyzed the data using the Student's T-test (TTEST) within the Microsoft Excel software program.

## Results

**Figure 1:**
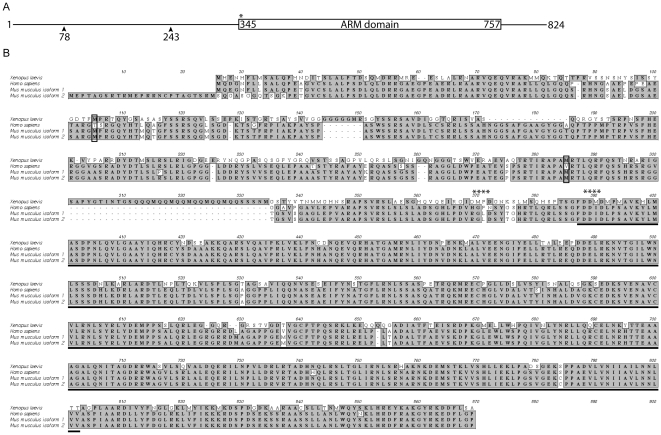
Pkp3 domain structure and amino acid sequence alignment. (A) Diagram of Pkp3 protein characteristics. Potential alternative translation initiation start sites are indicated with arrows, while a conserved, potential caspase cleavage site is labeled with an asterisk. (B) Sequence alignment of *Xenopus laevis,* human and two isoforms of mouse Pkp3. Identical and similar residues are indicated by grey highlighting. The Armadillo domain is underlined. Black boxes indicate methionines conserved with mouse that might serve as alternative translation initiation sites. Potential caspase cleavage sites are labeled with asterisks.

*Xenopus* Pkp3 shares strong similarity (71.2%) with human and mouse Pkp3 (Figure 1). The isoform isolated is encoded by 2472 base pairs, corresponding to 824 amino acids and a predicted protein molecular mass of 91 kDa. The sequence has been deposited online as Genbank ID: AF182522. Two potential alternative translational start sites were found in the predicted protein sequence that reside prior to the Armadillo domain and are conserved in mouse (but not human). In contrast to their known or putative relevance in p120-subfamily biology [3], [51], it is unclear at this time if alternative translational start sites are employed to generate different Pkp isoforms in vivo. Also found in Pkp3, as we recently characterized in some detail for delta-catenin [52], is a conserved predicted caspase cleavage site that if employed would generate ∼56 kDa and ∼35 kDa Pkp3 fragments. The amino-terminus is serine rich, accounting for approximately 15% of the residues in this region, and includes numerous potential phosphorylation sites. Overall, Xenopus Pkp3 resembles in its primary structure its mammalian counterparts, with caspase cleavage and phosphorylation being among possible points of regulation.

### *Xenopus* Pkp3 temporal expression

**Figure 2:**
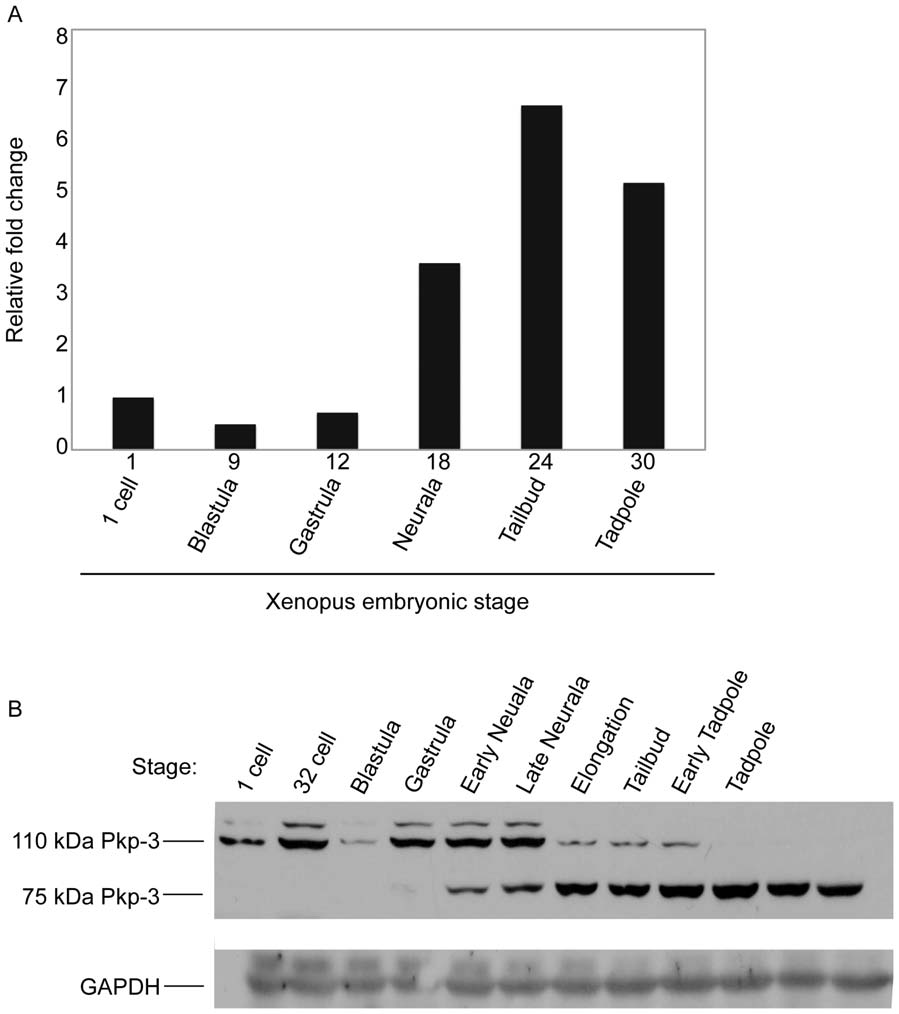
*Xenopus* Pkp3 temporal expression profiles. (A) Semi-quantitative real-time RT-PCR analyses indicating Pkp3 transcripts are deposited maternally at a relatively low level and then levels increase following gastrulation. (B) The 75 kDa Pkp3 protein isoform increases following gastrulation, while the 110 kDa isoform exhibits an inverse pattern. The polyclonal antibody generated against *Xenopus* Pkp3 amino acids 1–350 (N-terminal domain) recognizes Pkp3 protein products migrating at approximately 110 kDa and 75 kDa. Further, in early development, a fainter and more slowly migrating band is detected at approximately 120 kDa. Immuno-blot detection of GAPDH serves as a loading control.

To evaluate the temporal expression pattern of Pkp3, we performed semi-quantitative real-time RT-PCR utilizing total mRNA isolated from *Xenopus* embryos. Dissimilar from other characterized catenin mRNAs [26], [53], [54], [55], [56], *Xenopus* Pkp3 transcript levels appeared to increase rather dramatically (5–10 fold) following gastrula stages (Figure 2A), suggesting that Pkp3 may act predominately later in development. To examine *Xenopus* Pkp3 at the protein level, we generated a rabbit polyclonal antibody directed against its N-terminal domain (amino acids 1–350), that precedes the Armadillo domain (Figure 1A). The affinity-purified antibody recognizes two Pkp3 protein products migrating at approximately 110 kDa and 75 kDa, which are not detected using pre-immunization sera from the same rabbit (Figure S1A). The 110 kDa migrating product is larger than predictions based upon the isolated Pkp3 mRNA, suggesting that our cloning procedures may have missed a larger alternatively spliced mRNA, possibly one including a more upstream translation initiation site. Within the p120 subfamily, such alternative splicing is well known to take place across various catenin domains [26], [57], [58]. Finally, a third and larger protein product is occasionally (and weakly) detected at approximately 120 kDa. It is likely to be a Pkp3 isoform and may arise from an unknown alternative RNA splicing event, translation from a more upstream start site, or post-translational modification. With regards to Pkp3 protein levels, we found that consistent with the relative abundance of Pkp3 transcripts across developmental stages (Figure 2a), the 75 kDa translation product of Pkp3 increases following gastrulation. Complimenting the temporally increasing expression profile of 75 kDa Pkp3, the 110 kDa protein product is instead maternally deposited and expression decreases following gastrulation (Figure 2B). This suggests the possibility of distinct functions of the two isoforms according to developmental stage in amphibian development.

### *Xenopus* Pkp3 spatial expression

**Figure 3:**
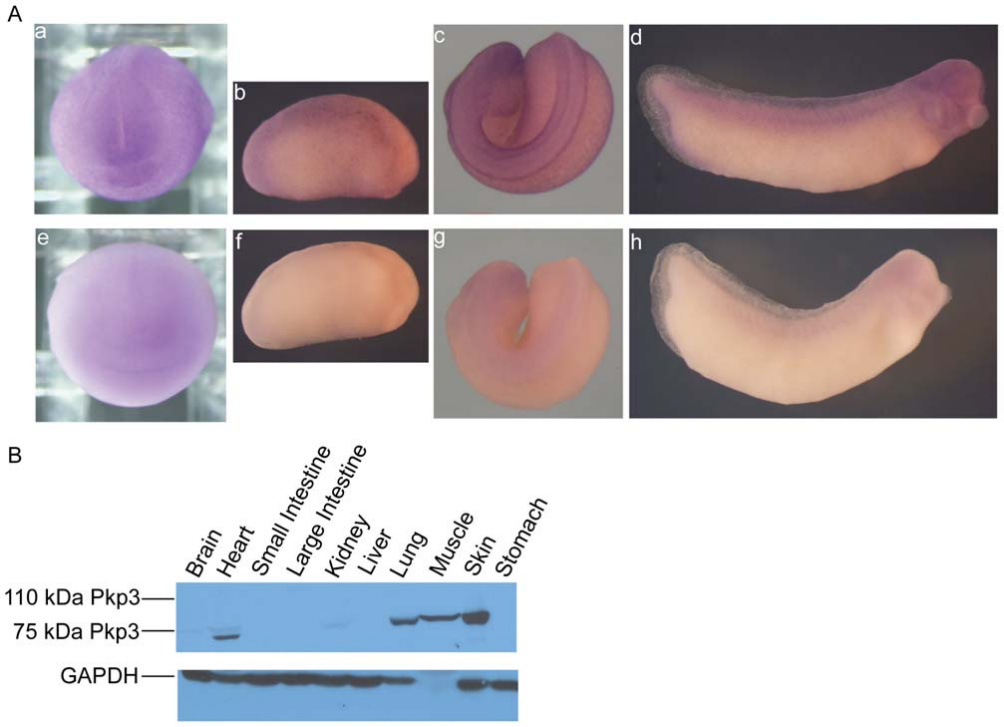
*Xenopus* Pkp3 spatial expression profiles. (A) Whole-mount in situ RNA hybridization reveals Pkp3 mRNA signals in the anterior and dorsal neural fold regions of neurula embryos (subpanel a). At elongation stages (stage 22; subpanel b), staining of the skin remains apparent, as does a concentration in dorsal structures. At tadpole stages, neural derived tissues including the brain, branchial arches and the spinal cord are stained, as are somites (subpanels c–d). As a basis for comparison (negative controls), we undertook sense-probe hybridizations in parallel (subpanels E–H). (B) Immuno-blotting on adult *Xenopus* tissue extracts. We used an affinity purified rabbit polyclonal antibody directed against the N-terminal domain of Pkp3, which clearly resolved the Pkp3 protein isoform migrating at approximately 75 kDa. This product was strongly expressed in heart, lung, muscle and skin. Weak, reproducible expression was detected in brain and kidney.

The spatial expression profile of Pkp3 was obtained through whole-mount in situ RNA hybridization of *Xenopus* embryos at various stages. We used a digoxigenin-labeled antisense full-length RNA probe of Pkp3 (Figure 3A a–d). A sense negative-control probe was hybridized and processed in parallel, and as expected did not produce significant signal intensities (Figure 3A e–h). In agreement with our qRT-PCR (Figure 2A), Pkp3 transcripts were less apparent prior to neurulation (data not shown), but subsequently become evident in the dorsal and anterior regions of neurula stage whole-mount embryos. At tailbud stages, embryos show staining in neural and neural crest derived tissues, as well as within somites and broad expression across the ectoderm. In particular, we observed signals consistent with the brain, eye vesicle and spinal cord, as well as the branchial arches. While quite faint, sectioning of these embryos confirmed Pkp3 expression in the ectoderm, branchial arches and neural tube (Figure S2 and data not shown).

To further examine the spatial profile of Pkp3 protein products, we performed immuno-blot analysis of adult *Xenopus laevis* tissues using our affinity purified polyclonal antibody. Consistent with Pkp3's predominant later expression in embryogenesis (Figure 2B), only the 75 kDa Pkp3 product was detected, being most prominent in tissues enriched for desmosomes, such as the heart, lung, muscle and skin (Figure 3B). Following longer exposures of the ECL films, Pkp3 was also clearly present in the kidney and brain (results not shown). Our findings indicate that *Xenopus* Pkp3 is most highly expressed at the mRNA and protein levels in desmosome-strengthened tissues, and is also found in tissues of neural derivation.

### *Xenopus* Pkp3 subcellular localization

**Figure 4:**
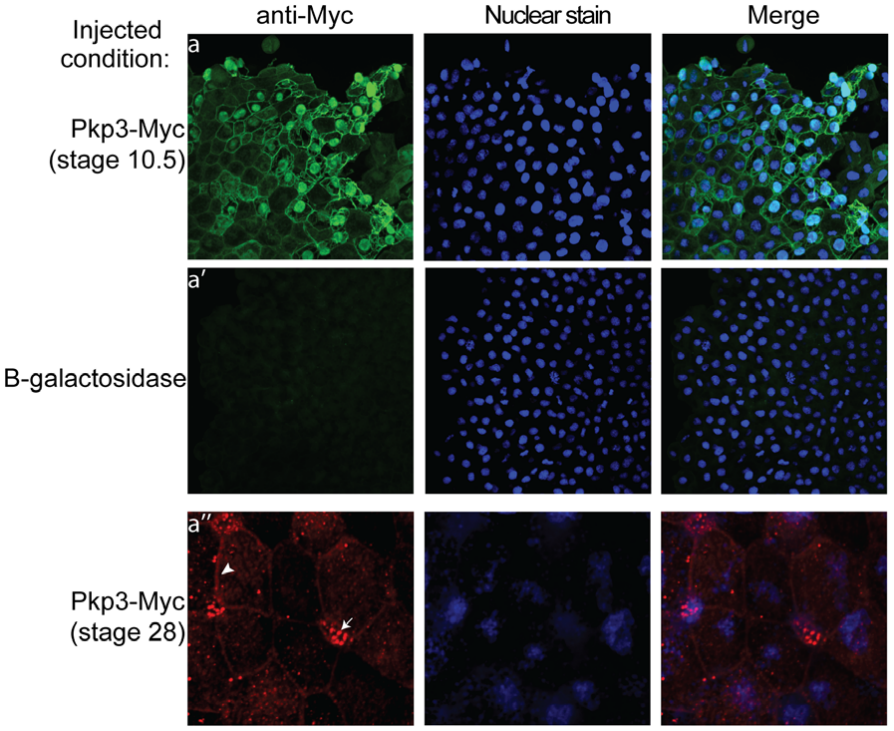
Pkp3 subcellular localization. Following earlier injection of exogenous Myc-tagged Pkp3 mRNA (0.5 ng injected into one-cell stage embryo), Pkp3 was visualized in the naïve ectoderm (animal cap) cells of blastula embryos at the plasma membrane, in the cytoplasm along what appear to be fibrous structures (possibly the intermediate filament network), and in the nuclear compartment (subpanel a). At late neurula stage the exogenous Pkp3 was again detected at the plasma membrane (arrowhead), in the nuclear compartment (though much weaker), and as punctate apically disposed spots (conceivably basal bodies or other cilia associated structures, arrow), in multiciliated cells intercalating upwards from the deeper sensoral layer (subpanel a″).

Catenin family members have been shown previously to localize to differing subcellular compartments, consistent with even a single catenin's varied functions [2], [4], [59]. This includes interactions with cadherin cytoplasmic tails at cell-cell junctions, modulation of small GTPases and protein translation in the cytoplasm, and alterations of gene expression in the nucleus. To assist in assessing Pkp3's subcellular localization, we generated a Myc-Pkp3 fusion construct, expressed it at levels similar to endogenous Pkp3 (Figure S1D) and examined its distribution in the *Xenopus* ectoderm of blastula stage embryos (animal caps), and late neurula stage embryos. At blastula stage, we found that Pkp3 is present at cell-cell borders, along fibrous cytoplasmic structures and in the nucleus (Figure 4a). In early tailbud, we consistently observed quite faint Pkp3 localization at cell borders, with even less intensity in the nucleus relative to blastula stages (Figure 4a″). Interestingly, we observed more intense punctate spots in select smaller cells within the same field that we later confirmed were within multiciliated cells intercalating from the sensoral layer of the ectoderm (data not shown). Nuclear localization of endogenous Pkp3 was likewise easily detected in uninjected embryos (Figure S1B), while cell border localization was considerably fainter to absent. This latter observation might arise from lesser accessibility of the added antibodies to the endogenous Pkp3 antigen(s) within mature desmosomal structures. This is, since Pkp3 is known to bind multiple desmosomal proteins [9], the exogenous protein might more easily decorate desmosomal structures in a manner that leaves the Myc-tag exposed for antibody based detection. In summary, our results suggest that *Xenopus* may prove a good model for studying Pkp3's junctional and/or cytosolic contributions to vertebrate development, and perhaps also its currently understudied nuclear functions.

### Knockdown of endogenous Pkp3 upon treatment with morpholinos

**Figure 5:**
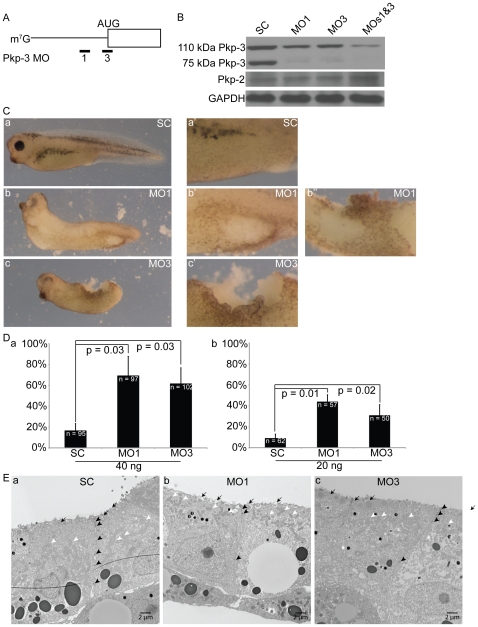
Depletion of endogenous Pkp3 results in skin fragility. (A) Diagram of morpholino-based depletion strategy. MO 3 targets the Pkp3 mRNA translational start site and MO 1 targets a non-overlapping upstream sequence in the 5′ UTR. Both morpholinos were designed to block translation initiation. (B) Immuno-blotting of hatching embryo extracts (stage 27) confirmed reductions of both Pkp3 protein isoforms following morpholino injection, but no reductions of Pkp2 protein (40 ng of each morpholino when injected individually or 20 ng of each morpholino when injected together into one-cell stage embryos). GAPDH serves as a loading control. (C) Injection of MOs 1 or 3 (40 ng into one-cell stage embryos) results in skin fragility (subpanels b and c, respectively), contributing to what can become lethal ectodermal damage sustained as the embryo hatches from the Vitelline membrane (higher magnification in subpanels b′, b″, and c′). Negative control embryos injected with standard control morpholino (40 ng) did not exhibit significant phenotypes (subpanels a-a′). (D) Quantification of the skin fragility effects are indicated in subpanel “d”, where P-values indicate statistical significance. As indicated, either 40 or 20 ng of each morpholino was injected into one-cell stage embryos. (E) Skin of tailbud stage embryos injected at one-cell stage with 40 ng standard control morpholino (subpanel a) compared to 40 ng MO 1 or 3 injected embryos (subpanels b and c, respectively). Pkp3 depleted ectoderm contains fewer basally disposed desmosomes (black arrow heads), while in apical regions desmosomes appear equally abundant. Note also that in skin of Pkp3 knockdown embryos, mitochondria have become more apically disposed (white arrowheads), and secretory vesicles have become much larger along the apical surface (black arrows).

The temporal and spatial expression of Pkp3, noted above, suggested that Pkp3 might contribute to various embryonic stages and processes. To assess this possibility, we designed antisense morpholinos to lower endogenous Pkp3 protein expression in *Xenopus*. Specifically, two independent oligonucleotide sequences were designed to compliment either the start ATG (MO3), or the proximal 5′ UTR (MO1) of the Pkp3 mRNA (Figure 5A). Reducing the probability of off-target effects, the morpholinos employed had no discernable complementation to the mRNA of *X. laevis* Pkp2. Further, while *X. laevis* Pkp1 was not resolved in the existing databases, *X. tropicalis* Pkp1 likewise lacked sequence complementation. To characterize their efficacy, the Pkp3 morpholinos were injected at varying doses (1–40 ng) into early cleavage stage embryos, which were then observed through tadpole stages. Immuno-blot assay of extracts obtained from embryos injected at the 1-cell stage confirmed that Pkp3 was reproducibly depleted by 40 ng of either MO1or MO3 or a mixture of the two morpholinos totaling 40 ng, but not by the standard control (SC) morpholino (Figure 5B). This knockdown demonstrates, the 110 and 75 kDa bands to be authentic (120 kDa band not observed/shown). Likewise, the translation of an exogenous Pkp3 mRNA, that included 90 bp of the 5′ UTR, was prevented upon the co-injection of either MO1 or MO3 (1 ng; Figure S1C). Of note, Pkp2 mRNA and protein levels were not affected by injection of mopholinos directed against Pkp3 (Figure 5B and S3).

### Pkp3 depletion results in ectodermal fragility

To assess the developmental impact of Pkp3 depletion, we injected MO1 or MO3, or both together into the animal pole of 1-cell embryos, or into a single blastomere of 2-cell embryos. Based upon gross external observation, these MO-injected embryos did not present any significant phenotypes until early tailbud stages, when hatching from their protective Vitelline envelope normally occurs. In contrast to SC injections (negative control), MO1 or MO3 injected embryos experienced damage to their surface ectoderm during shedding of the Vitelline membrane, which exerts tension and presumably frictional effects upon the hatching embryo. The phenotypic penetrance following Pkp3 depletion varied from 30–100%, as a function of the particular batch of embryos, amount of morpholino and the morpholino employed (Figure 5C–D). To obtain higher resolution views of the ectodermal phenotype, we turned to transmission electron microscopy. Following use of either of the Pkp3 targeting morpholinos, we observed reductions in the size and number of more basally localized desmosomal densities (Figure 5E and Figure S4A). Interestingly, while we do not presently understand the underlying basis, ectodermal cells of Pkp3 depleted embryos also displayed apically clustered mitochondria, as well as a significantly increased number and size of mucus secretory vesicles. Through the use of scanning electron microscopy (Figure S4B), the more roughened surface features of embryos depleted of Pkp3 (using combined morpholinos), appeared to reflect the increased number and sizes of underlying mucus secretory vesicles making contact and then fusing with the plasma membrane (compare right-hand panels of Figure S4B that shows pocked/roughened apical surface with panels b and c of Figure 5E, showing vesicles making surface contact). Additionally, scanning EM revealed defects in the motile cilia present upon multiciliated cells, as evident in Figure S4B. Such reductions in motile cilia were further reflected in lessened immuno-fluorescent intensity when staining for acetylated alpha-tubulin, which is commonly used to resolve cilia as well as established neurons (see panel a versus a′ of Figue 6D). Intriguingly, additional immuno-fluorescence analysis suggested that the multiciliated cells may themselves be reduced in number, or exhibit delays in intercalation upon Pkp3 depletion (data not shown). Finally, we noted that the very fine but observable surface demarcation of cell-cell borders within the ectoderm became less apparent following Pkp3 depletion (compare especially the lower panels of Figure S4B).

**Figure 6:**
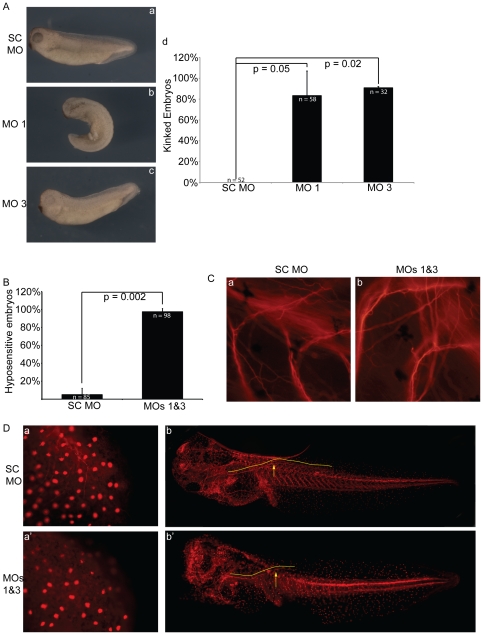
Pkp3 depletion results in tactile hyposensitivity and neural defects. (A) MO 1 or 3 injected embryos, that survive hatching have permanently kinked axes (subpanels b and c, respectively) (40 ng at one-cell stage). This is quantified in subpanel d, where the P-values indicate statistical significance. (B) Further observation revealed that Pkp3 depleted embryos were non-responsive to gentle tactile stimuli as observed by light shaking of their incubation dish, while controls attempted to escape from such stimuli. Upon more pronounced stimulation, it became evident that the embryos were able to swim normally, indicating retained muscle function (etc.) (data not shown). (C) Through the use of an established neural marker (acetylated alpha tubulin), examination of motoneurites arranged at somite boundaries revealed no large differences between controls and Pkp3 depleted embryos. (D) Neural processes present in the posterior region (tailbud) of control tailbud stage embryos (subpanel a), as well as a tadpole stage lateral neural tract of unknown identity (subpanels b), were reproducibly found to be less apparent and/or foreshortened following Pkp3 depletion (subpanels a′ and b′, respectively). Morpholinos (40 ng each) were injected at the one-cell stage.

To briefly survey another tissue dependent upon desmosome function, we looked at the heart. Here, we found that Pkp3 depleted embryos surviving ectoderm defects exhibited gross reductions in heart size as well as notable slowing of the beat rate at tadpole stages (Videos S1 and S2). In summary, a number of phenotypes are apparent in Xenopus embryos depleted of Pkp3. While future study will be needed, some outcomes may not be the direct result of altered desmosome function, such as the noted effects upon vesicle trafficking or cilia.

### Touch hyposensitivity and neural phenotypes in Pkp3 depleted embryos

Skin fragility was found at varying penetrance in Pkp3 knock down embryos as noted, yet more detailed observation further revealed that depleted embryos hatch from the Vitelline membrane at later developmental stages than do controls. Upon hatching, MO1 or MO3 injected embryos maintained a laterally curled appearance, apparently the result of their prolonged entrapment. With possible reasons for their extended entrapment discussed below, such embryos exhibited a fixed lateral curvature or kink along the anterior-posterior axis (Figure 6A). Interestingly, if the Vitelline membrane was manually removed at an earlier developmental stage to reduce embryo entrapment times, the penetrance of this phenotype was significantly lowered (Figure S5). A further obvious and unexpected phenotype was that depleted embryos, importantly including those with intact ectoderm, were not responsive to common laboratory stimuli such as gentle movement of their incubation dish or a light touch with a pipette tip (Figure 6B and data not shown). These observations suggested potential defects in neural or muscular development or function. Immuno-histochemical examination of the somites, early muscular structures, suggested that somite formation was not grossly altered (Figure S6A). A further functional indication of normal muscle capability was that embryos appeared to swim normally following more robust mechanical stimulation, such as from firm prodding with a pipette tip (data not shown). Sectioning followed by H&E staining revealed that somites appeared grossly normal and oriented properly (Figure S6B and data not shown). It should be noted that the knockdown embryos were generally more fragile in head regions during sectioning procedures, with neighboring tissues not remaining as closely associated. Likewise, while possibly arising during sectioning, the boundary between the notochord and neural tube was altered in a proportion of Pkp3 depleted embryos (Figure S6B subpanel d).

To examine potential effects of Pkp3 depletion on neural structures that conceivably would influence embryo touch-responsiveness or the execution of embryo hatching, we used an antibody commonly used to mark differentiated neurons (directed against acetylated alpha-tubulin). While bundled motor neurons running within the inter-somitic regions appear unaffected by either MO1 or MO3 (Figure 6C), we observed a loss of fine neural structures in the most posterior regions of early tailbud embryos. Additionally, we observed the consistent diminution in length of a lateral neural tract within tadpole stage embryos, which failed to extend into the tail region as far as those of SC injected controls (Figure 6D and Figure S7). Such altered neuronal morphologies may conceivably interfere with transducing or processing stimuli, possibly including neuro-muscular outputs to enable swimming or embryo hatching. Alternatively, or in addition, even small perturbations of the ectoderm may disrupt relationships between mechanosensory and signal transducing cells, thus having an impact upon responses such as touch.

### Pkp3 may have a role in neural crest cell induction and migration

**Figure 7:**
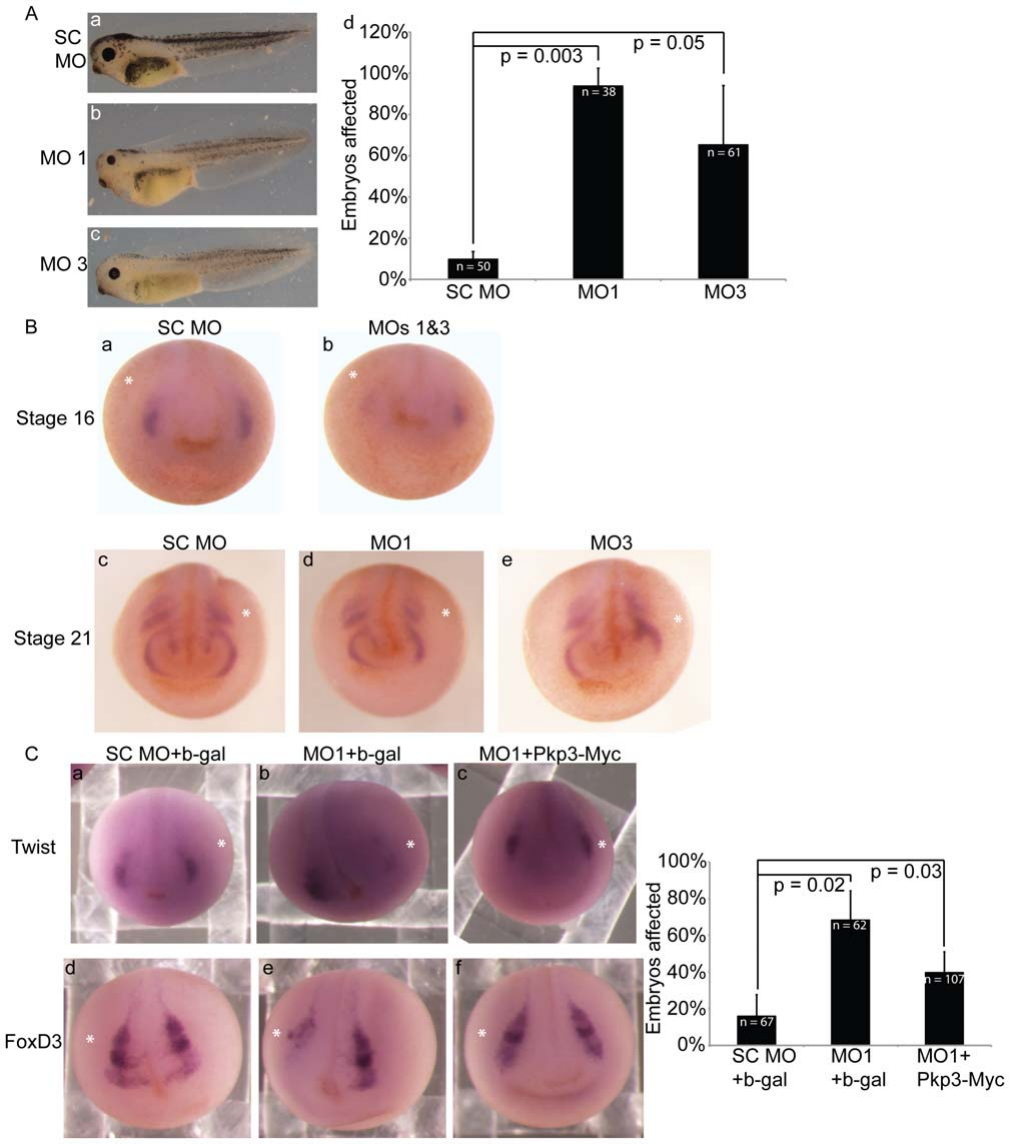
Neural crest induction and migration is disrupted upon Pkp3 depletion. (A) Injection of MO 1 or 3 resulted in loss of pigmentation and reduced eye size. 40 ng of either morpholino was injected at the one-cell stage. (B) In situ hybridization of the neural crest marker, Twist, following Pkp3 depletion (20 ng total MO into a single dorsal blastomere at the four-cell stage). At neurula stage 16, early (inductive) neural crest expression of Twist is reduced or lost (subpanel b). At neurula stage 21, later (migratory) neural crest expression likewise appears affected, as the tracts in Pkp3 depleted embryos do not extend (subpanel d–e) in the normal pattern (subpanel c). (C) Expression of the neural crest markers, Twist and FoxD3, was analyzed by in situ hybridization following knockdown of Pkp3. Pkp3 morphants showed reductions of both markers in the neural crest domain, that could be partially rescued upon co-injection of exogenous Pkp3 lacking the morpholino targeted sequence(s). 20 ng of each morpholino and 100 pg of either Myc-tagged beta-galactosidase or Myc-tagged Pkp3 mRNA was injected in a single dorsal blastomere. Quantification of these results is represented in the accompanying bar graph, where P-values indicate statistical significance.

To assess the impact of Pkp3 depletion within the prospective neuro-ectoderm, especially given Pkp3's expression pattern as reflected in Figures 3A and 3B, morpholino injections were made into a single dorsal blastomere at the 4-cell stage, with rhodamine dextran coinjected as a lineage tracer. At tadpole stages, these embryos exhibited reduced eye size (subpanels b and c of Figure 7A). In embryos injected at the one-cell stage and later analyzed via sectioning, large segments of the eye were similarly found to be malformed (Figure S6B subpanel e). Also seen in embryo whole mounts (Figure 7A), was a notable reduction in pigment cells throughout the embryo. As pigment cells are a neural crest derivative, Pkp3's possible contribution to neural crest was suggested. We thus tested an established marker of neural crest in embryos depleted of Pkp3 (versus SC injected). At early and late neurula stages (stages 16 versus 21; Figure 7B), reporting respectively on inductive and migratory processes, the depletion of Pkp3 significantly reduced expression of Twist. FoxD3, another established neural crest marker, likewise exhibited disrupted expression at late neurula stages in response to Pkp3 depletion. Suggesting the specificity of these observations, we partially rescued the expression of both neural crest markers upon coinjection of exogenous Pkp3 (mRNA lacking the morpholino binding site; Figure 7C), but not with coinjection of exogenous beta-galactosidase. Based upon the pigment-loss and peripheral nervous system phenotypes, as well as the initial marker analysis, it appears that Pkp3 may contribute to forming at least some neural crest derived tissues. As additional tissues likewise depend upon the neural crest, future work will examine effects upon the craniofacial skeleton as well as the heart outflow tract.

## Discussion

In vertebrates, diversification and expansion of catenin members derived from an ancient delta-catenin-like protein, populating the p120- and Pkp-subfamilies [3]. At cell-cell contact regions, the subfamilies preferentially localize to the adherens or desmosomal junction, respectively [60], [61], [62]. In the context of nuclear signaling, there are likely distinctions between the catenins, as best established for beta-catenin (binds the TCF/LEF transcriptional repressor) and p120 (binds the Kaiso transcriptional repressor) [63], [64], [65]. Many catenin roles have yet to be to elucidated, but briefly, catenins have been found to contribute to junctional structures, to the modulation of cytoplasmic small-GTPases (and translation, etc.), and to gene regulation [1], [5], [8], [21], [25], [27], [59], [63], [64], [66], [67], [68]. The Pkps were first characterized in association with desmosomal junctions, which provide physical integrity to mechanically stressed tissues, such as the heart and epidermis [4], [30], [31], [37], [69], [70], [71], [72], [73]. The Pkps may further transduce extracellular signals to the nucleus. Pkp3 is an Armadillo-domain protein that interacts with a range of proteins, remarkably including most identified desmosomal plaque components, several RNA binding proteins (PABPC1, FXR1, G3BP), and proteins involved in vesicle trafficking, such as Dynamin 1-like protein [9], [19], [40]. Whole animal targeting of mouse Pkp3 demonstrated its relevance to the prevention of skin inflammatory responses and the formation of desmosomes in certain epidermal cells [39]. Pkp3 removal was not embryonic lethal as had been observed in Pkp2 knockout mice [74]. Given Pkp3's wide expression in mice, and its multiple protein partners, the absence of a dramatic knockout phenotype was somewhat unexpected [39]. However, it was reasonably suggested to arise from compensatory mechanisms provided by Pkp1 and/or Pkp2, which bear >50% overall similarity, an analogous domain structure, and engage in some but not all of the same protein associations.

Using amphibian embryos, we together with independent investigators have found in a number of instances that a catenin's depletion generates more obvious phenotypes than does its corresponding knockout in mammals/mice. For example, there are more pronounced developmental effects following knockdown of Xenopus ARVCF- or delta-catenin than there is following either's knockout in mice [24], [26], [75] (R. Kucherlapati, personal communication). Therefore, to examine Pkp3 while taking advantage of the experimental advantages of the amphibian system [47], we applied knockdown approaches in *Xenopus laevis*.

In keeping with available sequence-tag data from mice, we found that Pkp3 mRNA levels increase in Xenopus following gastrulation (http://www.ncbi.nlm.nih.gov/UniGene/ESTProfileViewer.cgi?uglist=Mm.350037). This coincides with the appearance of a 75 kDa Pkp3 protein isoform that persists in the adult frog. Presumably due to maternal protein deposits made to the oocyte and passed onwards to the egg and embryo, we did not observe Pkp3 protein levels drop immediately following morpholino injection (data not shown). At such early stages (cleavage and blastula), we resolved a Pkp3 protein isoform migrating at 110 kDa, which showed reduced expression only after gastrulation, and no detectable presence in adult tissues. These results leave open the possibility that the two products have partially distinct functions in early versus later embryonic development. As is well known to occur for p120-catenin, the 110 and 75 kDa isoforms of Pkp3 might originate from alternative translation initiation sites [3], [51]. Alternatively, Pkp3 may be subject to caspase-3 (or other) cleavage, as we recently revealed for delta-catenin [52]. Although there exists a potential and conserved caspase-3 cleavage site in Pkp3, calculated to produce a ∼75 and ∼35 kDa fragments from the parental 110 kDa form, it awaits future evaluation. Whole-mount in situ and immunoblot analysis indicates that Pkp3 is present in the skin and lungs of *Xenopus* and absent from the liver, consistent with previous expression data obtained from mouse tissues and mammalian cell lines [30], [37], [40], [41], [42]. However, distinct from prior reports, we further observe Pkp3 localization in neural derivatives, heart, kidney and muscle, but not in the intestine or stomach. Interestingly, with some variance between species, it is has been shown that peripheral nerves and certain structures of the central nervous system are connected to surrounding sheaths of epitheloid cells via desmosome structures [76]. The closely related p120-catenin subfamily has been shown to have widespread expression in adult *Xenopus* tissues [26], [54], [55]. The more restricted although partially overlapping expression profile of Pkp3 suggests that it might fulfill specialized functions.

At the intracellular level, *Xenopus* Pkp3 localizes to a number of cell compartments including the nucleus, which had previously been ascribed as potentially exceptional or artifactural in mammalian systems [9]. However, our findings regarding Pkp3 are based upon both endogenous and exogenous detection, and coincide well with detailed descriptions of nuclear Pkp1 and Pkp2, as well as multiple other catenin family members [31], [32], [36], [52], [77]. Indeed, we have preliminarily resolved a number of nuclear proteins in association with Pkp3 that are now being characterized (data not shown). Plasma membrane-associated Pkp3 in *Xenopus* is expected to contribute to desmosome formation and/or stability, as has been well characterized in mammalian systems [9], [30], [37], [39], [46]. The presence of Pkp3 at desmosomal junctions, as well as in the cytoplasm and nucleus, leaves open the possibility that this catenin assists in cross-talk between junctional and cytoplasmic and/or nuclear compartments, in conjunction with assisting in a structural capacity at desmosomes. Conceivable Pkp3 signaling roles might be akin to those of beta- and p120-catenin, that are present at adherens junctions but also participate in nuclear and cytoplasmic processes. This might, for example, include Wnt signaling and small GTPase modulation [21], [66], [78], [79].

To examine Pkp3 functions in vivo, we used antisense morpholinos to block ribosome binding and thus translational initiation from the Pkp3 transcript. This standard depletion approach revealed a number of consistent phenotypic outcomes, which were evident using either of two distinct sequence specific morpholinos. Interestingly, in Pkp3 knockout mice, Pkps 1 and 2 were upregulated most likely as a compensatory mechanism [39], while in Xenopus we determined that Pkp2 mRNA and protein levels were not notably altered upon Pkp3 depletion. Thus, less pronounced compensation among Pkps in amphibians might partially account for the more severe effects we observed following Pkp3 knockdown in Xenopus.

In evaluating partially targeted tissues such as neural crest, where only one cell within a 4-cell embryo was injected with morpholino, the specificity of the Pkp3 depletion phenotype was supported using an add-back approach, where consistent fractional self-rescues were observed. As is often observed and would be expected, self-rescues were more difficult to obtain in embryos injected with morpholinos at the 1-cell stage (or both cells of a 2-cell embryo), since essentially the entire embryo must be coordinately rescued. In such cases, we employed the standard in the field, which was to reproducibly obtain similar phenotypes using independent (non-overlapping) morpholinos.

MO 1, directed upstream of the translational start site, exhibited the highest penetrance. Each morpholino caused skin fragility, heart deformation and hyposensitivity to touch following a delayed escape from the Vitelline membrane. Increased skin fragility was accompanied by a decrease in the number and size of basally disposed desmosomes within the ectoderm, consistent with prior Pkp3 depletion studies, and studies of patients with Pkp1 mutations that exhibit skin fragility ectodermal-dysplasia [39], [69], [80], [81]. Mutations or loss of desmosomal components, including Pkp2, are known to compromise desmosome structure and lead to arrhythmogenic right ventricular dysplasia and heart failure [74], [82], [83]. Our results in Xenopus likewise suggest that Pkp3 is needed in heart formation and/or maintenance in *Xenopus*, possibly participating in a desmosomal context similar to that of Pkp2 in human heart, and/or conceivably as an indirect consequence of effects upon neural crest, which contributes to the heart outflow tract [84].

Additionally, punctate Pkp3 localization in the same apical region where cilia are organized within multiciliated cells of the ectoderm, as well defects in the cilia of these cells following Pkp3 depletion, suggests a novel role for Pkp3 in cilia establishment or maintenance. Interestingly, we are now characterizing a number of intracellular trafficking and motor proteins that we have preliminarily identified in association with Pkp3, that were found previously by others to participate in cilia formation, cilia activity, vesicle transport or mitochondrial localization (data not shown). Consistent with potential Pkp3 contributions to vesicle transport, the structurally related Pkp2 was recently found in mammalian cells to affect kinesin dependent intracellular transport of Dsg2 [85].

We further uncovered an unexpected role for Pkp3 in amphibian sensitivity to tactile stimuli. The observed hyposensitivity that followed Pkp3 depletion may be due to the reduction or compromised function of certain sensory (and/or motor?) neural tracts along the length of the animal. To our knowledge, the requirement of Pkps in neural function has not been previously noted, and might prove to be an interesting area for future investigation. Tactile sensory pathways are partly derived from neural crest cells [86], [87], where we found indications that Pkp3 plays a necessary role. The neural crest gene expression markers (Twist & FoxD3), as well as neural crest derived pigment cells, were each notably reduced following Pkp3 depletion. Several signaling pathways act in neural crest cell specification and migration, including the canonical and noncanonical Wnt pathways [86], [88]. While speculative, since canonical Wnt signals are transduced by beta-catenin, with recent evidence also indicating the involvement of vertebrate p120-catenin [79], more distant members of the catenin family such as Pkp3 may additionally contribute to Wnt mediated processes in neural crest.

Thus, in our *Xenopus* studies, Pkp3 depletion led to a number of phenotypes. This is in contrast to the whole mouse targeted knockout that exhibited ectodermal defects, but no obvious additional phenotypes. As noted earlier, these species distinctions might be in part attributable to differing Pkp3 spatial expression patterns, including those in relation to Pkp1 and Pkp2, which may compensate to a greater extent for Pkp3 loss in mice [39]. Our results indicate that the depletion of Pkp3 has an impact upon multiple tissues, including skin, heart and neuronal tracts. Also, it seems that Pkp3 might ultimately prove to have more easily discernable roles (if any) in the nucleus of amphibians, as we have consistently detected nuclear Pkp3 whereas in mice this localization is less reproducible. A final possibility to consider is that Pkp3 may have evolved to exhibit at least some functional distinctions between amphibians and mammals, given that the Pkp-subfamily was recently characterized as evolving quickly, even relative to the related p120-catenin subfamily [3].

In summary, we have revealed essential roles for Pkp3 in amphibian development. Given the neurological as well as ectodermal phenotypes observed in this vertebrate, it may ultimately prove worthwhile to test for Pkp3 defects in human patients exhibiting neurological or ectodermal dysfunctions.

## Supporting Information

Figure S1 Characterization of affinity purified rabbit polyclonal antibodies that are directed against the N-terminal domain of Pkp3 (amino acids 1–350); and an additional demonstration of the efficacy of MO1 and MO3 in targeting Pkp3. (A) Immuno-blotting using the noted anti-Pkp3 antibodies specifically revealed 110 kDa and 75 kDa protein products in Xenopus embryo extracts (stage 25; also detected using whole sera). The same bands are not resolved using serum collected from the same rabbit prior to immunization. (B) Immuno-fluorescence detection of endogenous Pkp3, using the antibody noted above. Pkp3 is detected in the nucleus of non-dividing cells within blastula-stage ectoderm (animal caps), while the secondary antibody alone did not produce a detectable signal (nor did additional negative controls; data not shown). (C) Immuno-blotting of blastula extracts confirmed the reduced presence of exogenous HA-tagged Pkp3 protein following earlier co-injection (one-cell stage) of Pkp3 mRNA (250 pg) with even low-doses of MO1 or MO3 (1 ng of either morpholino). A non-specific band serves as a loading control. (D) Immuno-blots of early neurula and tailbud extracts, showing the level of exogenous Myc-tagged Pkp3 relative to endogenous Pkp3 (500 pg Pkp3 mRNA injection at one-cell stage).
(TIF)

Figure S2 Pkp3 tissue localization. Whole mount anti-sense in situ staining of tadpole stage embryos, followed by cross-sectioning and agarose embedding, reveals faint Pkp3 signals in the neural tube (arrowhead) relative to the sense (negative) control stained embryos. NT, neural tube; NO, notochord.
(TIF)

Figure S3 Pkp3 depletion does not notably affect Pkp2 mRNA levels. Following depletion of Pkp3 (40 ng MO1 or MO3 into one-cell stage embryos) RT-PCR was preformed on cDNA derived from stage 27 embryos. No significant change was detected in Pkp2 mRNA levels, relative to embryos injected with standard control (SC) morpholino. Pkp2 bands were quantified and normalized relative to the ODC loading control, with no statistically significant differences found.
(TIF)

Figure S4 Pkp3 depletion alters the appearance and localization of desmosome structures, as well as the morphology of additional cellular features. (A) High-magnification transmitted electron microscopy revealed that the depletion of Pkp3 (subpanels MO1 and MO3) results in reduced desmosome size and appearance. For example, MO3 injected embryos exhibit some desmosome-like structures where equivalent densities are not always properly paired between contacting cells (see arrowheads). (B) Scanning electron microscopy revealed altered ectoderm surface features in Pkp3 depleted embryos. For example, whereas in control embryos fine demarcations can be seen upon close examination to reflect the borders between cells, such demarcations are more difficult to discern in Pkp3 depleted embryos. Further, we observed increases in the size of what we expect are mucus secretory vesicles (pits on the surface), a finding that was likewise reflected in transmission electron micrographs (see Figure 4D). Defects in the cilia of multiciliated cells were also apparent in Pkp3 morphants. 40 ng of each morpholino was injected at the one-cell stage.
(TIF)

Figure S5 Pkp3 knockdown kink phenotype is a result of prolonged entrapment in the Vitelline membrane. The kinked phenotype of embryos injected with MO 1 or MO3 was significantly rescued upon manual removal of the Vitelline membrane at neurula stage 19. Normally, the embryo hatches later through the Vitelline membrane, at stages 33/34. 40 ng of each morpholino was injected at the one-cell stage. P-value indicates statistical significance.
(TIF)

Figure S6 Pkp3 depletion does not appear to affect early muscle formation. (A) Following injection with either standard control morpholino or a mix of MOs 1 and 3, the somites, an early muscle structure, do not appear disrupted. Note, the Pkp3 depleted embryo is laterally bowed towards the viewer, giving the false impression of compression of the somites. The number of somite segments was counted and compared, with no statistically significant differences found. 40 ng of each morpholino was injected at the one-cell stage. (B) Sectioning and histological staining of Pkp3 knockdown embryos reveals no defects in main body somites (subpanel b, SC embryo image obtained from second series of sectioning and staining). However, defects in the head regions were observed in some cases. While possibly arising during sectioning due to reduced tissue integrity, there appeared to be overlaps in some embryos of the notochord and neural tube, as well as eye defects (subpanels d and e). NT, neural tube; NO, notochord; SO, somite.
(TIF)

Figure S7 Pkp3 knockdown significantly affects certain peripheral neural structures. Neural processes present in the posterior region (tailbud) of control tailbud stage embryos were reproducibly found to be less apparent and/or foreshortened following Pkp3 depletion. Quantification of these results is shown here, where P-values indicate statistical significance. 40 ng of each morpholino was injected at the one-cell stage.
(TIF)

Video S1 SC morpholino injected embryo. Live imaging of *Xenopus* tadpole injected with 40 ng of SC morpholino at 1 cell stage. Note rapidly beating heart.
(MOV)

Video S2 Pkp3 knockdown embryo. Live imaging of Pkp3 MOs 1 and 3 injected embryo (20 ng each). Reduced heart size and slowed rate of heart beat compared to Video 1 are observed.
(MOV)
