# Supplementary figures and images for: Plakophilin-3 Is Required for Late Embryonic Amphibian Development, Exhibiting Roles in Ectodermal and Neural Tissues
Source: PLoS One. 2012 Apr 5;7(4):e34342. doi: 10.1371/journal.pone.0034342 (PMC3320641; doi:10.1371/journal.pone.0034342)

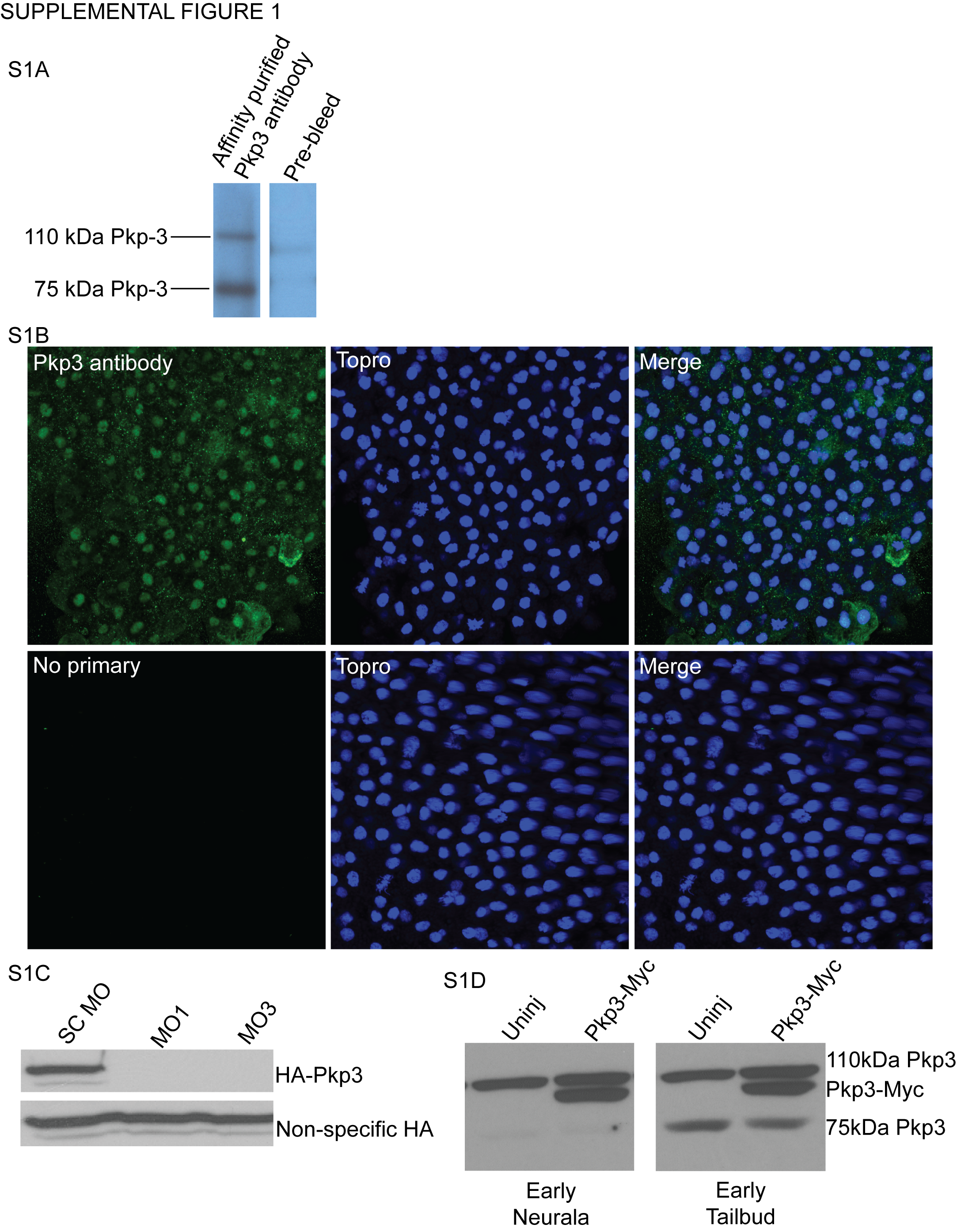

Supplement: Figure S1 — Characterization of affinity purified rabbit polyclonal antibodies that are directed against the N-terminal domain of Pkp3 (amino acids 1–350); and an additional demonstration of the efficacy of MO1 and MO3 in targeting Pkp3. (A) Immuno-blotting using the noted anti-Pkp3 antibodies specifically revealed 110 kDa and 75 kDa protein products in Xenopus embryo extracts (stage 25; also detected using whole sera). The same bands are not resolved using serum collected from the same rabbit prior to immunization. (B) Immuno-fluorescence detection of endogenous Pkp3, using the antibody noted above. Pkp3 is detected in the nucleus of non-dividing cells within blastula-stage ectoderm (animal caps), while the secondary antibody alone did not produce a detectable signal (nor did additional negative controls; data not shown). (C) Immuno-blotting of blastula extracts confirmed the reduced presence of exogenous HA-tagged Pkp3 protein following earlier co-injection (one-cell stage) of Pkp3 mRNA (250 pg) with even low-doses of MO1 or MO3 (1 ng of either morpholino). A non-specific band serves as a loading control. (D) Immuno-blots of early neurula and tailbud extracts, showing the level of exogenous Myc-tagged Pkp3 relative to endogenous Pkp3 (500 pg Pkp3 mRNA injection at one-cell stage). (TIF) [file pone.0034342.s001.tif]

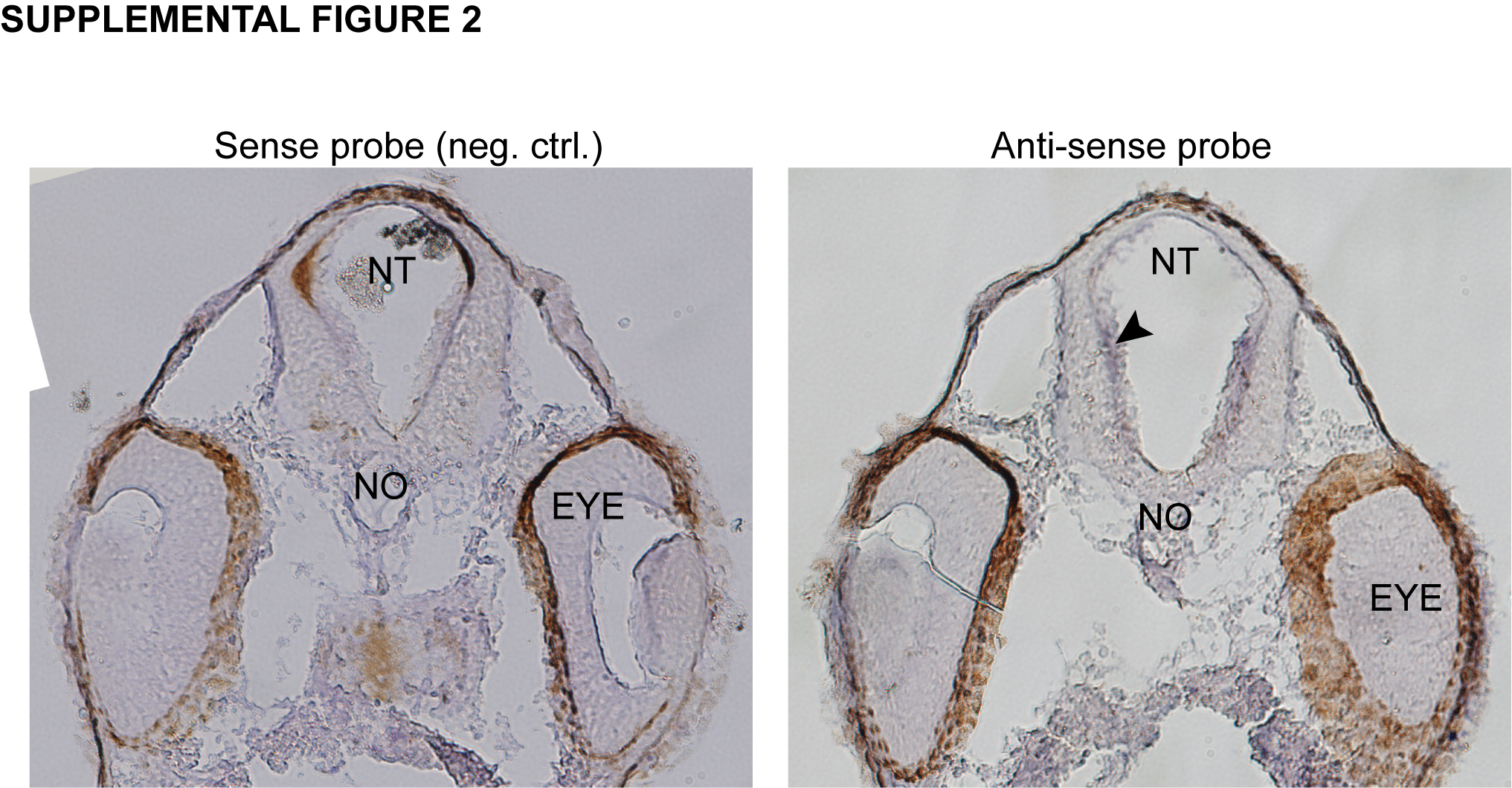

Supplement: Figure S2 — Pkp3 tissue localization. Whole mount anti-sense in situ staining of tadpole stage embryos, followed by cross-sectioning and agarose embedding, reveals faint Pkp3 signals in the neural tube (arrowhead) relative to the sense (negative) control stained embryos. NT, neural tube; NO, notochord. (TIF) [file pone.0034342.s002.tif]

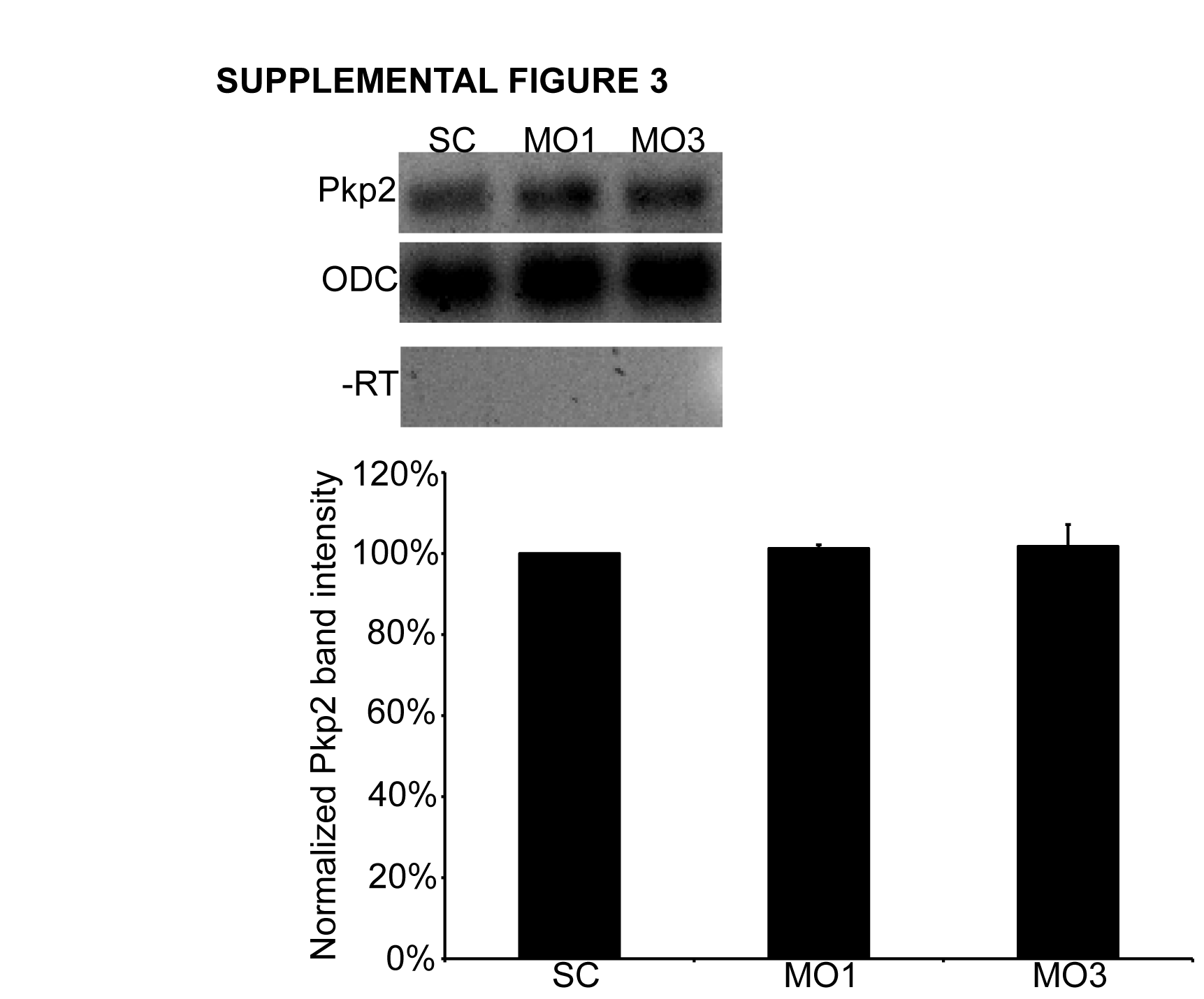

Supplement: Figure S3 — Pkp3 depletion does not notably affect Pkp2 mRNA levels. Following depletion of Pkp3 (40 ng MO1 or MO3 into one-cell stage embryos) RT-PCR was preformed on cDNA derived from stage 27 embryos. No significant change was detected in Pkp2 mRNA levels, relative to embryos injected with standard control (SC) morpholino. Pkp2 bands were quantified and normalized relative to the ODC loading control, with no statistically significant differences found. (TIF) [file pone.0034342.s003.tif]

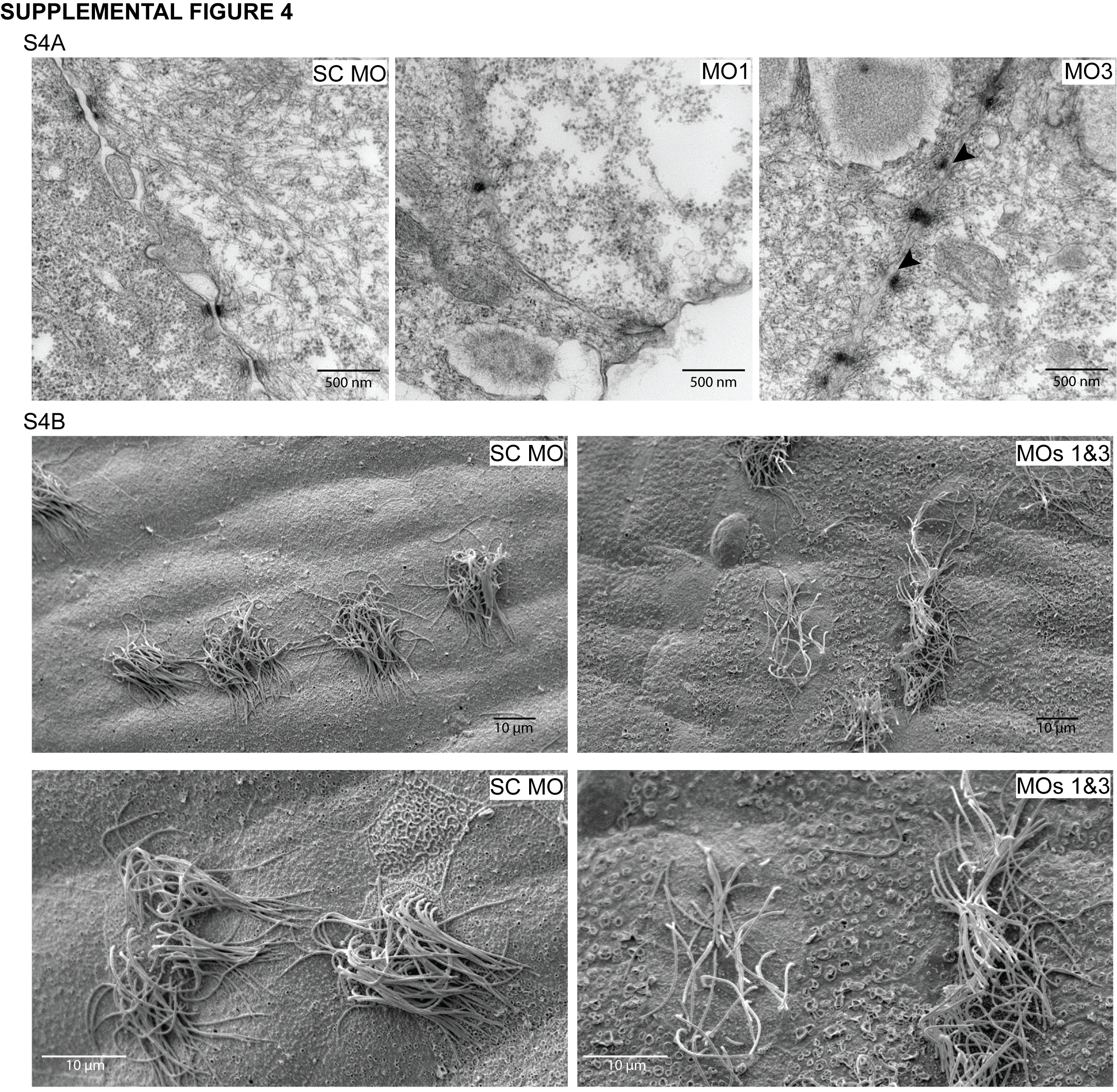

Supplement: Figure S4 — Pkp3 depletion alters the appearance and localization of desmosome structures, as well as the morphology of additional cellular features. (A) High-magnification transmitted electron microscopy revealed that the depletion of Pkp3 (subpanels MO1 and MO3) results in reduced desmosome size and appearance. For example, MO3 injected embryos exhibit some desmosome-like structures where equivalent densities are not always properly paired between contacting cells (see arrowheads). (B) Scanning electron microscopy revealed altered ectoderm surface features in Pkp3 depleted embryos. For example, whereas in control embryos fine demarcations can be seen upon close examination to reflect the borders between cells, such demarcations are more difficult to discern in Pkp3 depleted embryos. Further, we observed increases in the size of what we expect are mucus secretory vesicles (pits on the surface), a finding that was likewise reflected in transmission electron micrographs (see Figure 4D). Defects in the cilia of multiciliated cells were also apparent in Pkp3 morphants. 40 ng of each morpholino was injected at the one-cell stage. (TIF) [file pone.0034342.s004.tif]

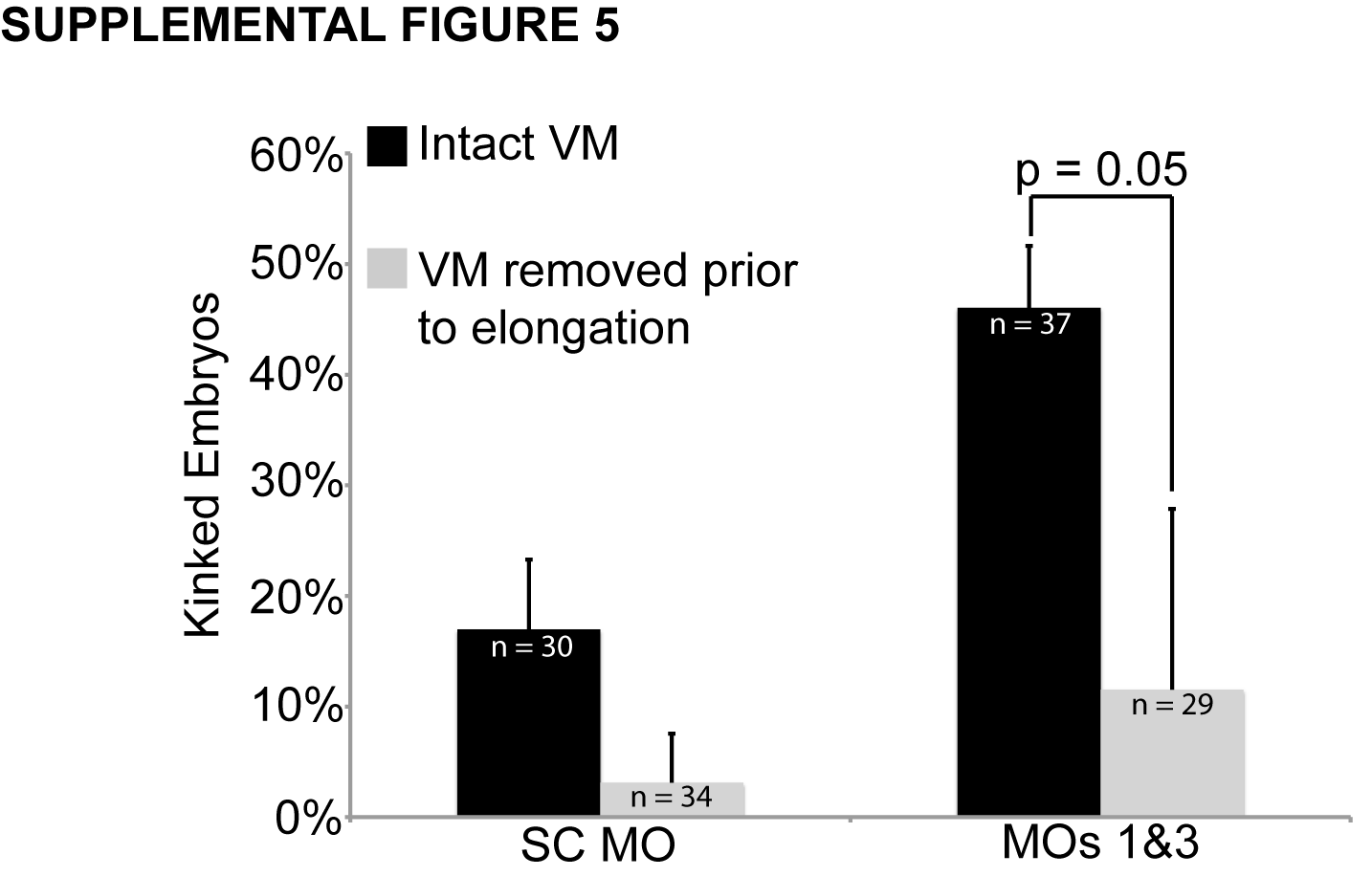

Supplement: Figure S5 — Pkp3 knockdown kink phenotype is a result of prolonged entrapment in the Vitelline membrane. The kinked phenotype of embryos injected with MO 1 or MO3 was significantly rescued upon manual removal of the Vitelline membrane at neurula stage 19. Normally, the embryo hatches later through the Vitelline membrane, at stages 33/34. 40 ng of each morpholino was injected at the one-cell stage. P-value indicates statistical significance. (TIF) [file pone.0034342.s005.tif]

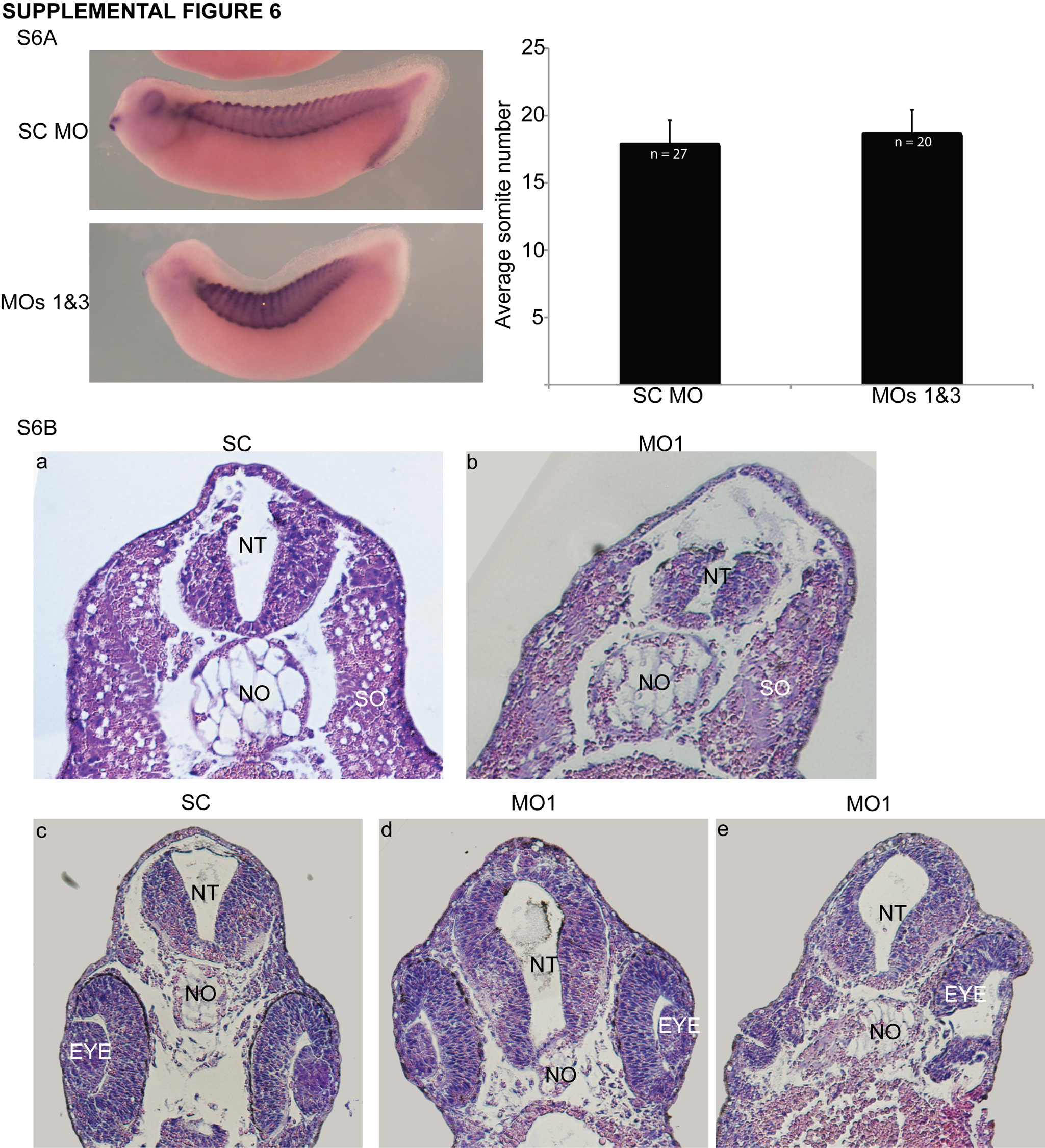

Supplement: Figure S6 — Pkp3 depletion does not appear to affect early muscle formation. (A) Following injection with either standard control morpholino or a mix of MOs 1 and 3, the somites, an early muscle structure, do not appear disrupted. Note, the Pkp3 depleted embryo is laterally bowed towards the viewer, giving the false impression of compression of the somites. The number of somite segments was counted and compared, with no statistically significant differences found. 40 ng of each morpholino was injected at the one-cell stage. (B) Sectioning and histological staining of Pkp3 knockdown embryos reveals no defects in main body somites (subpanel b, SC embryo image obtained from second series of sectioning and staining). However, defects in the head regions were observed in some cases. While possibly arising during sectioning due to reduced tissue integrity, there appeared to be overlaps in some embryos of the notochord and neural tube, as well as eye defects (subpanels d and e). NT, neural tube; NO, notochord; SO, somite. (TIF) [file pone.0034342.s006.tif]

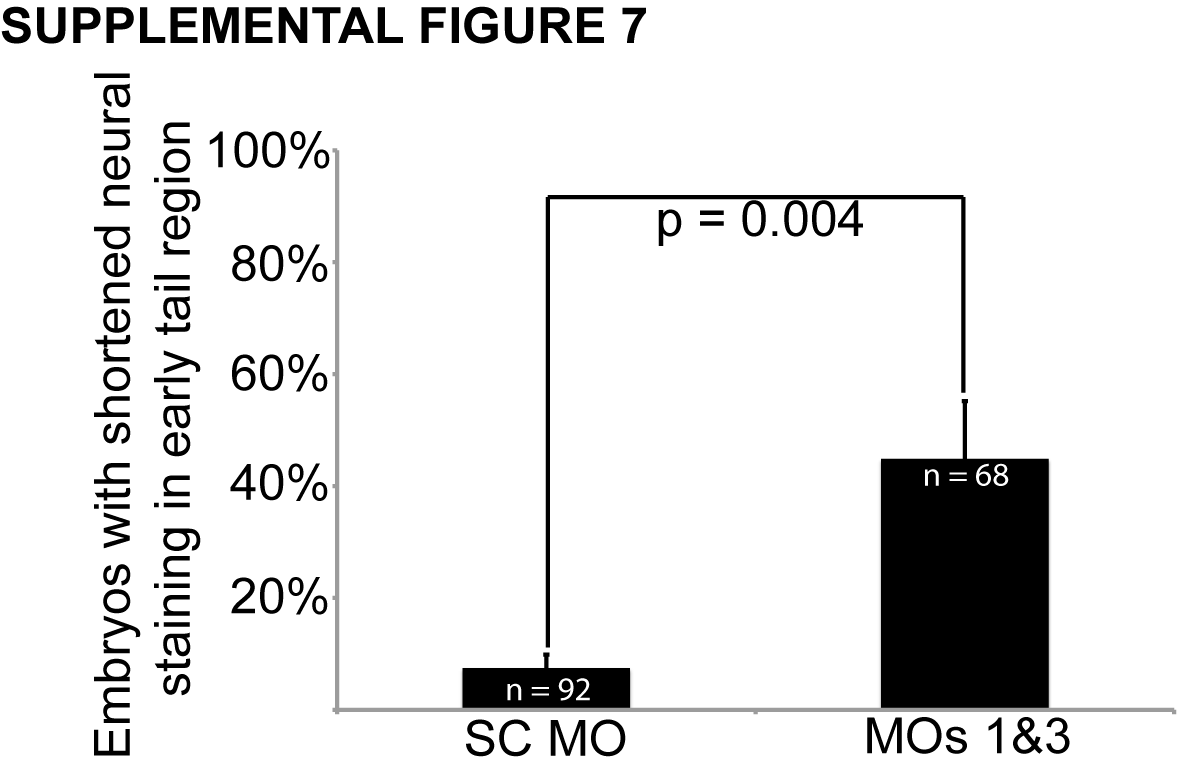

Supplement: Figure S7 — Pkp3 knockdown significantly affects certain peripheral neural structures. Neural processes present in the posterior region (tailbud) of control tailbud stage embryos were reproducibly found to be less apparent and/or foreshortened following Pkp3 depletion. Quantification of these results is shown here, where P-values indicate statistical significance. 40 ng of each morpholino was injected at the one-cell stage. (TIF) [file pone.0034342.s007.tif]
